# Efficacy and mechanism of cyprosulfamide in alleviating the phytotoxicity of clomazone residue on maize seedling

**DOI:** 10.3389/fpls.2024.1512055

**Published:** 2024-12-20

**Authors:** Lanlan Sun, Chen Zhang, Hongle Xu, Wangcang Su, Fei Xue, Qiuli Leng, Yujia Niu, Chuantao Lu, Renhai Wu

**Affiliations:** ^1^ Institute of Plant Protection, Henan Academy of Agricultural Sciences, Zhengzhou, China; ^2^ Henan Key Laboratory of Agricultural Pest Monitoring and Control, Zhengzhou, China; ^3^ Key Laboratory for Integrated Crop Pests Management on Crops in Southern Region of North China, Zhengzhou, China

**Keywords:** antioxidation, carotenoid, gene expression, Clomazone residue, phytotoxicity

## Abstract

**Introduction:**

The residues of clomazone (Clo) can lead to phytotoxic symptoms such as foliar bleaching, reduced plant height, and decreased maize yields. Herbicide safener represent one of the most economically efficient strategies for mitigating herbicide-induced damage.

**Methods:**

In this study, various seed treatments were implemented, including the immersion of maize seeds in water (CK), immersion in Cyprosulfamide (CSA), soil supplemented with clomazone (ClO) and CSA+ClO, evaluated physiological indicators, chlorophyll content, and qRT-PCR analyses of the maize plants were evaluated under the different treatments.

**Results and discussion:**

The objective of this study was to investigate the impact of CSA on mitigating residual damage caused by Clo on maize and elucidate its mechanism. Compared to the CK, treatment with Clo resulted in significant inhibition of maize plant height, fresh weight, chlorophyll content, and carotenoid levels by 19.0%, 29.9%, 92.5%, and 86.3% respectively. On the other hand, under CSA+Clo treatment, milder inhibition was observed with reductions of only 9.4% in plant height and 7.2% in fresh weight, as well as decreases of 35.7% and 21.8% respectively in chlorophyll and carotenoid contents. The findings revealed that the application of CSA effectively mitigated the inhibitory effects of Clo residues on maize plant height, fresh weight, carotenoids and chlorophyll content. Additionally, the combination of CSA and Clo reduced MDA levels by 13.4%, increased SOD activity by 9.7% and GST activity by 26.7%, while elevating GSSG content by 31.3% compared to Clo alone, ultimately mitigating oxidative damage in maize plants. qRT-PCR analysis showed that the expression of five P450 genes (*CYP72A5, CYP81A4, CYP81Q32, CYP81A9, CYP81A36*), nine GST genes (*GST30, GST31, GSTIV, GSTVI, GST21, GST7, GST37, GST25, IN2-1*), and two UGT genes (*UGT76C2, UGT83A1*) significantly high increased by 6.74-, 10.27-, 4.98-, 10.56-, 25.67-, 16.70-, 46.92-,7.53-, 5.10-, 238.82-, 143.50-, 4.58-, 31.51-, 39.3-, 4.20-, 10.47-fold after CSA+Clo treatment compared to that in the Clo treatment. The pre-treatment of CSA led to the upregulation of five P450 genes, nine GST genes, and two UGT genes, which may be associated with the metabolism of Clo in maize. Overall, this study suggests that CSA could be effectively mitigates Clo residual damage by up-regulating detoxification-related genes, enhancing chlorophyll content and activities of antioxidant enzymes.

## 1 Introduction

Clomazone (Clo), an isoxazolane herbicide, was primarily employed for the control of annual grass and broadleaf weeds in crops such as peanuts, soybeans, and oilseed rape through pre- and post-emergence applications (Chang et al., 2022). So far, there are over 200 clomazone-based products in China, mostly used for soil treatment (http://www.chinapesticide.org.cn/, accessed 1 March, 2024). The applied clomazone do not completely degrade over time, with a major portion remaining in the soil as residues (Li et al., 2024). The half-life of clomazone in soil ranges from 15 to 157 days, and its biological activity lasts for over 180 days, making it a persistent environmental contaminant (Li et al., 2015; Nalini et al., 2016; Liu et al., 2023). For example, the Mollisols region of China’s cropland soils contained 56 detected herbicides, with residues ranging from 0.31 to 1558.13 μg/kg. Clomazone exhibited the highest concentrations (Li et al., 2024). The high solubility and low log Kow (2.55) of clomazone make it easily absorbed by subsequent sensitive crops, causing phytotoxic symptoms like foliar bleaching, reduced plant height, and decreased yields (Ferhatoglu and Barrett, 2006; Golden et al., 2017; Wang et al., 2021; Arana et al., 2023; Cao et al., 2024; Liu et al., 2024). Therefore, it is essential to assess the mitigation of residual damage caused by Clo in maize for the safe production of maize.

Safeners are chemical agents added to herbicide formulations to protect crops from potential harm without reducing the effectiveness of the herbicides against targeted weeds, allowing farmers to apply them more safely and effectively while minimizing crop injury and achieving desired crop protection (Siddique, 2023). The number of safener utilized in commercial herbicide formulations currently stands at approximately 20. Safeners are commonly applied as seed treatments (e.g., Naphthalic anhydride, Cyometrinil, Oxabetrinil, Fluxofenim, Flurazole in maize or sorghum), water surface applications (Dymron, Cumyluron, Dimepiperate in rice), or spray formulations (Benoxacor, Furilazole, AD-67, Fenclorim used for pre-emergence in maize; Cloquintocet-mexyl, Fenchlorazole-ethyl, Mefenpyr-diethyl, Isoxadifen-ethyl used for post-emergence in cereals or maize) (Rosinger, 2014). Cyprosulfamide (CSA) was launched by Bayer CropScience AG in 2009 and is the only safener that can be used for pre- and post-emergence herbicides in maize (Sun et al., 2023). It can effectively mitigate damage to maize caused by isoxaflutole (Gerhards and Santel, 2020), thiencarbazone-methyl (Zheng et al., 2015), fenpyrazone (Lian et al., 2023), and nicosulfuron (Sun et al., 2023). The majority of studies have focused on the efficacy of CSA in alleviating herbicide phytotoxicity, with limited research conducted on residual herbicide damage.

The previous research has shown that safeners effectively reduce the negative impacts of herbicide residues. N-tosyloxazolidine-3-carboxamide derivatives, compound 9 (N-phenoxyacety-2-methyl-2,4-diethyl-1,3-oxazolidine) showed significant protection against tribenuron-methyl and nicosulfuron via enhancing the glutathione (GSH) content and glutathione S-transferase (GST) activity (Ye et al., 2019; Zhang et al., 2021). The tolerance of crops is enhanced by safeners through two primary mechanisms. First, the amount of herbicide reaching the active site may be reduced or its interaction at the target site may be disrupted (Riechers et al., 2010). Second, the activation of detoxifying enzymes by safeners plays a crucial role in the metabolism of herbicides (Jia et al., 2023). The focus of most studies on detoxifying enzymes was glutathione S-transferases (GSTs), which catalyze the conjugation of herbicides with endogenous glutathione in wheat, maize (Sun et al., 2018), and rice (Hu et al., 2020). Other classes of detoxifying enzymes, such as cytochrome P450s (CYP450s), UDP-dependent glycosyltransferases (UGTs) (Zhao et al., 2023), ATP-binding cassette transporters (ABC transporters) (Yang et al., 2018) and antioxidant system (SOD, POD, CAT) (Baek et al., 2019; Hu et al., 2020, 2021), are also involved in the process of detoxification. The previous investigation have confirmed that CSA effectively alleviate the damage caused by nicosulfuron on maize plants under adverse environmental conditions, such as high temperatures and dry weather, while simultaneously enhancing herbicidal efficacy against weeds (Sun et al., 2016). The mitigation of nicosulfuron damage was associated with the enhancement of nicosulfuron metabolism through the up-regulation of genes involved in the detoxification pathway (Sun et al., 2023). We propose that these metabolic enzyme genes play a crucial role in the mitigation of Clo toxicity by CSA.

The present study objectives to investigate mitigating effect and the mechanism of the mitigation of Clo residual damage by CSA. The compounds known as CSA and Clo are both biologically active xenobiotic substances. As herbicides can induce peroxidation in crops, the impact of safeners on ROS in maize remains uncertain. The effects of CSA on maize physiology and the expression of metabolic enzyme genes in maize under the influence of CSA and Clo are systematically studied. Here, we aim to (1) investigate the potential of CSA in enhancing chlorophyll and GST activity, (2) explore the antioxidant enzyme activities in maize seedlings under Clo and CSA treatment and (3) identify genes closely related to the mode of action of the CSA and Clo metabolic pathways. This will address the issue of residual damage caused by Clo. It also provides a theoretical framework for investigating the mechanism of CSA. The combined use of these methods not only improves crop protection and yields, but also helps to make agricultural practices more sustainable by reducing environmental risks.

## 2 Materials and methods

### 2.1 Plant material and growth conditions

The soil for the test was loamy soil from the experimental base of Henan Academy of Agricultural Sciences, and the physical and chemical properties of the soil are shown in **Table 1**. The maize seed used for the test crop was Zhengdan 958, purchased from Henan Qiule Seeds Technology,Co.,Ltd, Henan Province, China. The herbicide safener CSA with 94% purity was purchased from Hebei Lansheng Biotech Co., LTd, Hebei Province, China. Clomazone (360 g L^-1^) was produced by Yunfa Chemical Factory (Shanghai, China) Co.

**Table 1:** Physical and chemical properties of soil.

Soil type	Ph	Organic matter/(g/kg)	Available nitrogen/(mg/kg)	Available phosphorus/(mg/kg)	Available potassium/(mg/kg)	Total nitrogen/(g/kg)
Soil	8.38	5.75	110.53	17.84	213.44	0.50

Maize seeds were soaked in distilled water for 2 h, followed by germination at a temperature of 28°C in the dark for 48h. when the radicle length of maize reached approximately 0.5cm, the maize were subjected to immersion in water (CK) or 120 mg/L CSA solutions for 2 h. Afterward, the seeds were washed three times with distilled water. Subsequently, they were planted in 7 × 8 cm (diameter × height) square nutrient pots, with five seeds per pot. The pots contained soil supplemented with Clo at a concentration of 4 mg/kg (the concentration selection process is given in the **Supplementary Data Sheet**). Seeds were sown to a depth of 3 cm. All pots were incubated in an artificial greenhouse (20 to 25°C, L//D = 12 h//12 h, and 80% relative humidity). Five biological replicates were performed for each treatment. Plant height and fresh weight were measured 7 days after seedling emergence. The shoots of maize under different treatment were collected 7 days after emergence for chlorophyll, malondialdehyde (MDA), GST, reduced glutathione (GSH), oxidized glutathione (GSSG), antioxidant activity analyze and qRT-PCR determination.

### 2.2 Physiological indicators and chlorophyll content determination

Total chlorophyll (chlorophyll a, chlorophyll b, carotenoid and total chlorophyll) was extracted by 80% acetone and the content assays was detected using spectrophotometric methods (Qi et al., 2023). GST, GSH and GSSG were extracted and quantified using determined by a kit (Grace, Suzhou, China) in accordance with the manufacturer’s instructions. Protein content was determined by Bradford’s koji method (Bradford, 1976). The crude enzyme solution for determined SOD and CAT activity was extracted in accordance with the methodology proposed by Nayeri et al. (2023). SOD enzyme activity was determined by the nitroblue tetrazole (NBT) method (Zhang et al., 2022); CAT activity was determined by UV absorption (Nafi U et al., 2022). The MDA content was determined by thiobarbituric acid colorimetric method (Yu et al., 2022).

### 2.3 RNA extraction and real-time fluorescence quantitative PCR assay

The transcriptom data published by Sun et al. (2023) was utilized to identify a total of twenty-eight (**Supplementary Table S1**) maize detoxification genes that were up-regulated in response to CSA treatment. Based on the respective gene sequences, candidate gene-specific primers were designed using Primer Premier 5.0 and synthesized by Sangon Biotech CO., Ltd. (primers are shown in **Supplementary Table S2**). Leaf tissue samples stored at -80°C were ground in liquid nitrogen and total RNA was extracted using Vazyme reagent (catalog No. RC411-01; Vazyme Biotech Co., Ltd., Nanjing, China) according to the manufacturer’s instructions. Quantitative real-time quantitative polymerase Chain reaction amplification templates were 1 μL (3000 ng μL^-1^) of cDNA, 0.4 μL (10 μM) of each primer, 5 μ of TB Green premix Ex TaqII (Takara, Beijing, China), and 3.2 μL of distilled water. According to our previous methods, *EF1α* was used as a reference gene (Sun et al., 2018). CK was used as the control tissue, and the relative quantification of the gene in each tissue was determined by the comparative 2^-ΔΔCT^ method.

### 2.4 Statistical analysis of data

The data are presented as mean ± standard deviations. The significance of the treatment differences was assessed using one-way analysis of variance (ANOVA) based on the least significant difference (LSD) at a significance level of P < 0.05 level, employing the SPSS software 26.0. The graphs were plotted using GraphPad Prism 9.

## 3 Results

### 3.1 The protective effect of CSA on maize against Clo residue injury

**Figure 1:**
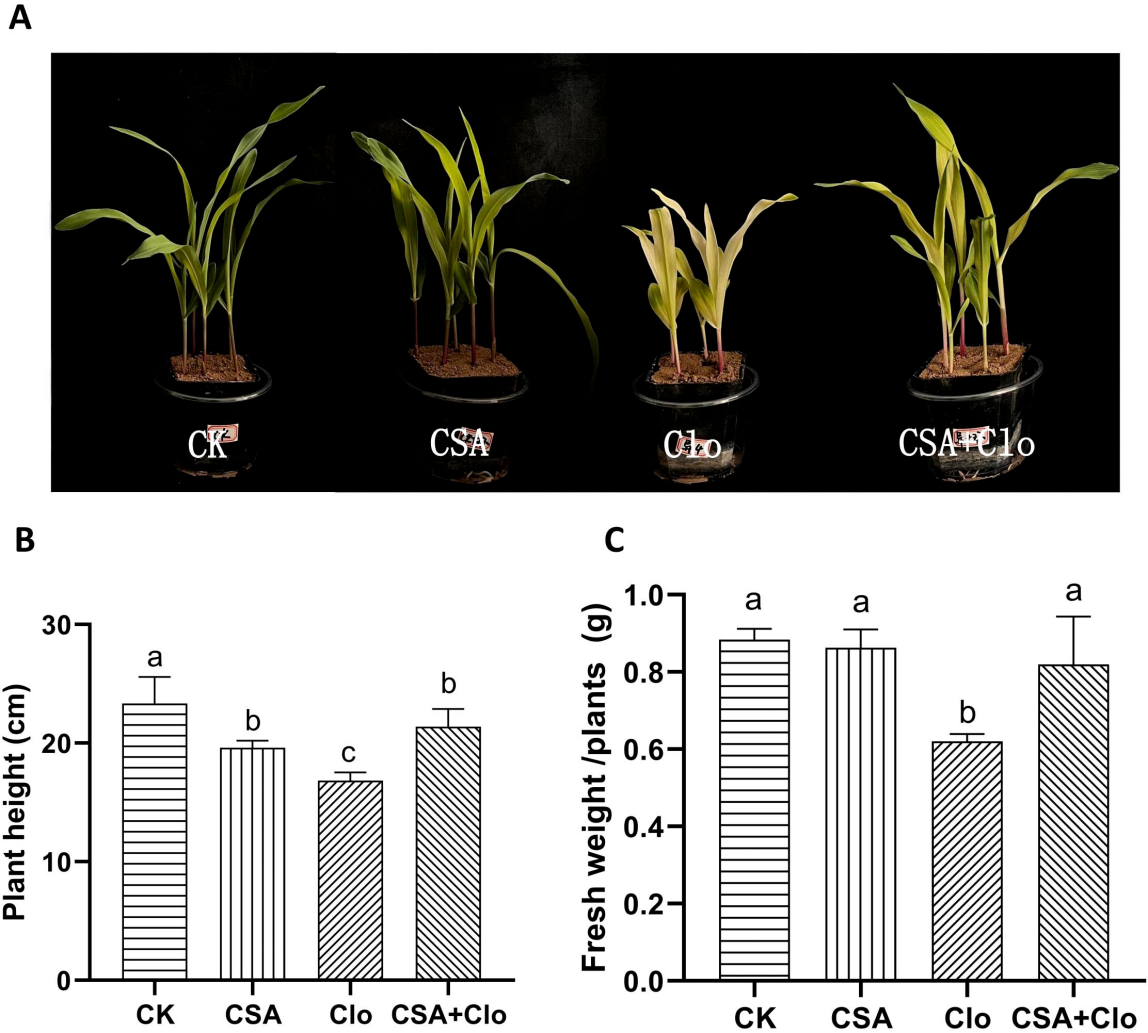
Effect of clomazone (Clo) and Cyprosulfamide (CSA) on the growth level of maize. Morphological responses of maize plants under control (CK), Clo (the residual concentration of Clo in the soil was 4 mg/kg), CSA (the maize were soaked in 120 mg/L CSA solutions for 2 h) and CSA+Clo treatments and grown for 7 days after seedling emergence **(A)**. Effect of Clo and CSA on the plant height **(B)**. Effect of Clo and CSA on the fresh weight **(C)**. The bars represent the averages ± standard deviations of five replicates. Lowercase letters indicate significant differences within each treatment in maize plant height and fresh weight, respectively (*p* < 0.05), determined by Duncan test.

The economic crop maize, often rotated with soybeans, frequently suffers from clomazone residue-induced injury (Li et al., 2014). The toxicity of Clo in maize was investigated under both CSA-treated and non-treated conditions. After 7d of treatment, the visible symptoms of Clo include leaf chlorosis, wilting, and inhibited plant growth (**Figure 1A**). Maize plants showed a 19% inhibition in plant height and a 29.9% reduction in fresh weight (**Figures 1B, C**). However, the CSA-pretreatment significantly protected maize from Clo exposure and suppressed its growth inhibition. The bleached maize leaves started to regain their green color (**Figure 1A**). The inhibition of plant height and fresh weight of maize was reduced by 9.4% and 7.2% (**Figures 1B, C**). The CSA-pretreatment alone did not significantly affect maize fresh weight. The results confirmed that enhancing Clo detoxification in maize plants correlated with a protective effect, which depended on soaking CSA 2 hours before Clo treatment.

### 3.2 Enhanced chlorophyll content in maize leaves after pre-treatment with CSA

**Figure 2:**
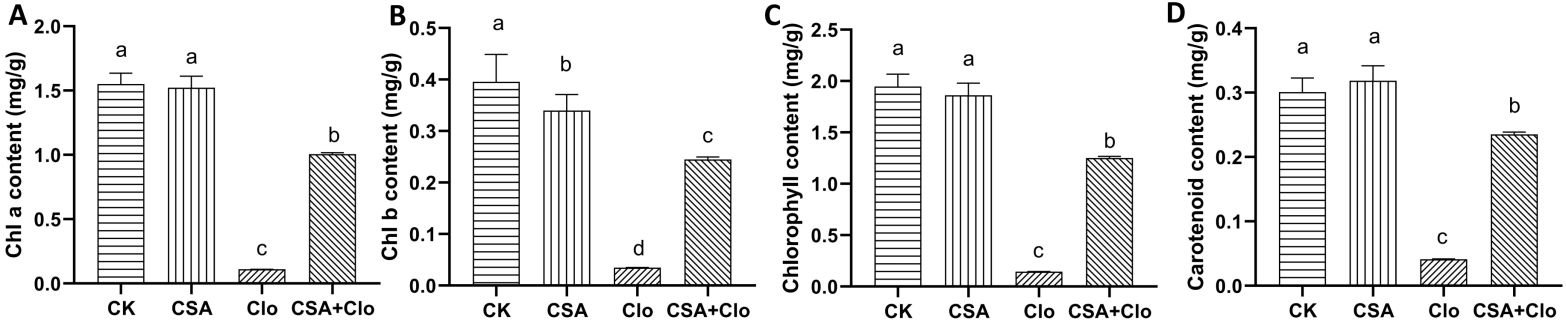
The content of Chlorophyll a **(A)**, Chlorophyll b **(B)**, total Chlorophyll content **(C)** and carotenoid **(D)** in different treatments. The bars represent the averages ± standard deviations of five replicates. Lowercase letters indicate significant differences within each treatment in maize plants (*p* < 0.05), determined by Duncan test.

Clo inhibits 1-deoxy-D-xylulose-5-phosphate (DXP) synthase in the first committed step of the non-mevalonate isoprenoid pathway in plastids, causing impaired chloroplast development and loss of pigments including carotenoids in susceptible plants (Ferhatoglu and Barrett, 2006). To assess the impact of CSA on maize leaf chlorophyll content, the chlorophyll and carotenoid content in maize leaves was determined following CSA treatment. The content of chlorophyll a, chlorophyll b, total chlorophyll and carotenoid of maizes in CSA-treated maize leaves show no significant effect compared to those in the control treatment (**Figures 2A–D**). The content of chlorophyll a, chlorophyll b, total chlorophyll and carotenoid in maize plants decreased by 92.9%, 91.2%, 92.5% and 87.0% respectively under Clo treatment compared to the control (**Figure 2**). This may be related to the mechanism of Clo. The treatment with soaking CSA significantly mitigated the impact of Clo on chlorophyll a, chlorophyll b, total chlorophyll and carotenoid, resulting in an increase in content by 8.09, 6.05, 7.62 and 4.69 times, respectively. These results suggest that the pre-treatment with CSA may be increased the rate of Clo metabolism in maize plants.

### 3.3 Pre-treatment with CSA decreased MDA content and enhanced SOD and CAT activity

**Figure 3:**
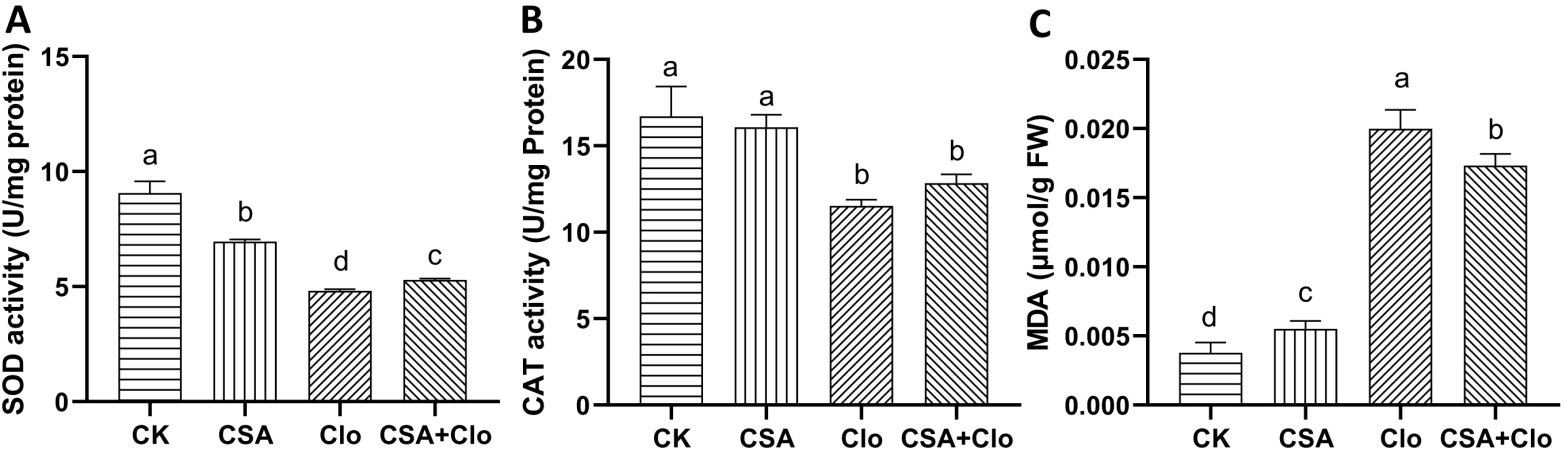
The effects of different treatments on the activities of SOD **(A)**、CAT **(B)** and contents of MDA **(C)**. The bars represent the averages ± standard deviations of five replicates. Lowercase letters indicate significant differences within each treatment in maize plants, respectively (*p* < 0.05), determined by Duncan test.

The activity of antioxidant enzymes has been shown to be associated with herbicide tolerance in various plants (Hu et al., 2020). To assess cellular damage induced by Clo and CSA, MDA content in maize plants was quantified. As shown in **Figure 3C**, The MDA content of maize increased significantly by 5.3 times with Clo treatment alone compared to the control. However, CSA+Clo treatment reduced the MDA content by 13.44% compared to Clo treatment alone. The result suggests that CSA may potentially mitigate the accumulation of MDA induced by Clo.

The Clo and CSA treatments resulted in alterations to in superoxide dismutase (SOD) and catalase (CAT) activities in maize (**Figures 3A, B**). The activity of SOD and CAT decreased by 46.80% and 31.04%, respectively, under Clo treatment compared to the control. However, when CSA+Clo treatment was applied, SOD and CAT activities increased by 9.7% and 11.4%, respectively, compared to Clo treatment alone (**Figure 3**). The capacity of the addition of CSA to reduce the level of MDA may be attributed to its potential to enhance the activities of the enzymes SOD and CAT.

### 3.4 Pre-treatment with CSA increased GSSG content and enhanced GST activity

**Figure 4:**
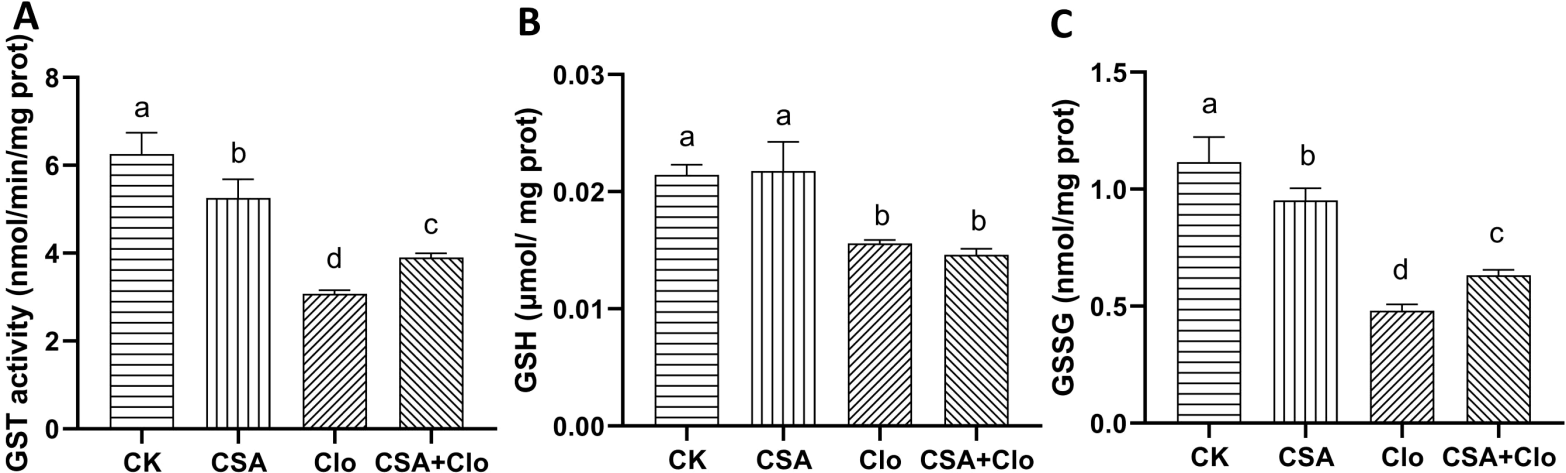
The effects of different treatments on the activities of GST **(A)** and contents of GSH **(B)**, GSSG **(C)**. The bars represent the averages ± standard deviations of five replicates. Lowercase letters indicate significant differences within each treatment in maize plants (*p* < 0.05), determined by Duncan test.

The enzyme GST plays a crucial role in helping crops detoxify herbicides by facilitating the binding of GSH with various types of herbicides (Rosinger, 2014). The Clo residue treatment significantly inhibited GST activity and decreased GSH and GSSG content in maize seedlings. Specifically, there was a reduction of 50.80% in GST activity, 27.27% in GSH content, and 77.13% in GSSG content compared to the control group. However, this inhibition was alleviated after CSA+Clo treatement, with GST activity and GSSG content increasing by 26.7% and 31.3% compared to Clo treatment alone (**Figure 4**). GSH content was no significant effect between the Clo and Clo+CSA treatment. The results indicate that CSA enhances detoxification and metabolism of Clo in maize seedlings, improving their tolerance to Clo.

### 3.5 Metabolizing enzyme genes expression

**Figure 5:**
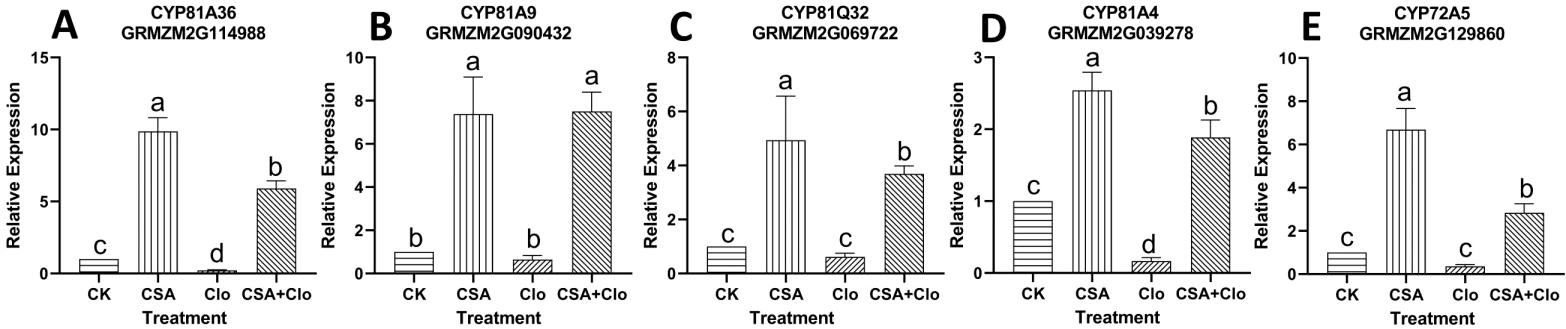
**(A)** CYP81A36 relative expression level; **(B)** CYP81A9 relative expression level; **(C)** CYP81Q32 relative expression level; **(D)**CYP81A4 relative expression level; **(E)** CYP72A5 relative expression level. qRT-PCR validation was performed to confirm five *P450* genes induced by Clo and CSA in maize. The bars represent the averages ± SD of five replicates. Lowercase letters indicate significant differences within each treatment in maize plants (*p* < 0.05), determined by Duncan test.

**Figure 6:**
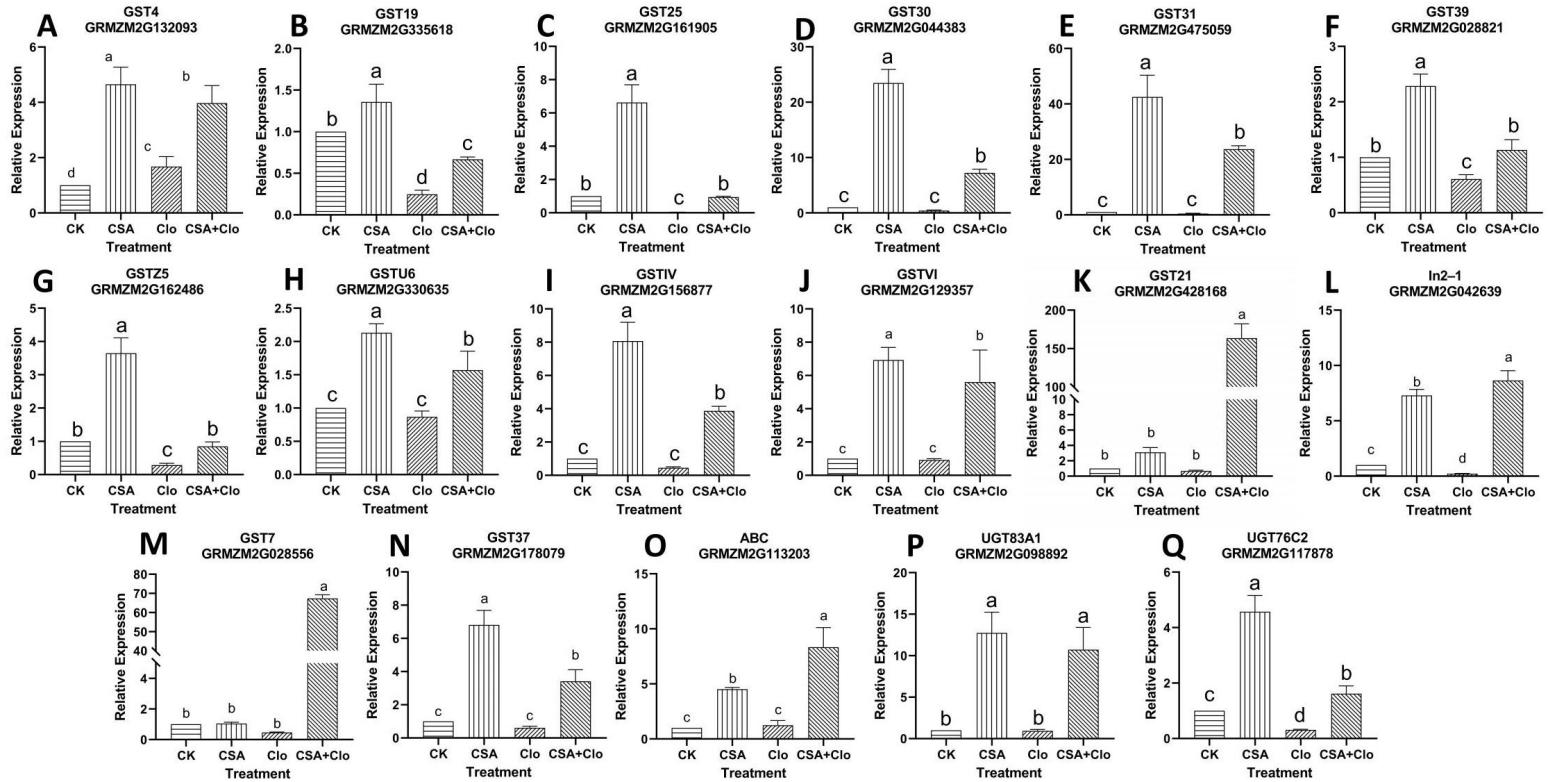
**(A)** GST4 relative expression level; **(B)** GST19 relative expression level; **(C)** GST25 relative expression level; **(D)** GST30 relative expression level; **(E)** GST31 relative expression level; **(F)** GST39 relative expression level; **(G)** GSTZ5 relative expression level; **(H)** GSTU6 relative expression level; **(I)** GSTIV relative expression level; **(J)** GSTVI relative expression level; **(K)** GST21 relative expression level; **(L)** In2-1 relative expression level; **(M)** GST7 relative expression level **(N)** GST37 relative expression level; **(O)** ABC transporter relative expression level; **(P)** UGT83A1 relative expression level; **(Q)** UGT76C2 relative expression level. qRT-PCR validation was performed to confirm nine *GST* genes, one *ABC* gene and two *UGT* genes induced by Clo and CSA in maize. The bars represent mean ± SD from five biological replicates. Lowercase letters indicate significant differences within each treatment in maize plants (*p* < 0.05), determined by Duncan test.

The expression of specific genes encoding detoxification enzymes in crops can be induced by safeners (Riechers et al., 2010). We previously identified several detoxification enzymes genes in maize induced by CSA through transcriptome (Sun et al., 2023). These CSA-upregulated genes are predicted to play a crucial role in mitigating the residual injury of Clo. Suitable primers were designed for 24 out of the 28 CSA-induced metabolizing enzyme genes, but *CYP72A14* and *UGT88A1* showed down-regulation in the CSA treatment, which contradicted the transcriptome data (refer to **Supplementary File** for details), leading to their exclusion. Finally, 22 candidate genes were screened, namely, *CYP72A5*, *CYP81A4*, *CYP81Q32*, *CYP81A9*, *CYP81A36*, *UGT83A1*, *UGT88A1*, *UGT76C2*, *ABC*, *GSTIV*, *GST30*, *GST19*, *GSTZ5*, *GST31*, *GST4*, *GST25*, *GST39*, *GST21*, *GST37*, *In2-1*, *GSTVI*, *GST7* and *GSTU6*. The expression levels of all 22 candidate genes were significantly up-regulated after CSA treatment alone, ranging from 1.05 to 42.52 in relative expression (**Figures 5**, **6**). The result aligns with the findings reported by Sun et al. (2023). However, the responses of these 22 candidate genes varied under Clo and CSA+Clo treatments.

As shown in **Figures 5B, C, E**, the expression levels of three P450 genes (*CYP81A9, CYP81Q32*, and *CYP72A5*) remained unchanged under Clo treatment. However, when treated with CSA+Clo, the expression of these three genes significantly increased by 10.56-, 4.98-, 6.74-fold compared to that in the Clo treatment. On the other hand, the relative expression level of *CYP81A36* and *CYP81A4* were 0.22 and 0.17, respectively, under the Clo treatment (**Figures 5A, D**). Their expression levels increased remarkably by 25.67- and 10.27- fold respectively under Clo+CSA treatment in comparison to Clo treatment.

As shown in **Figure 6**, *GST30*, *GST31*, *GSTU6*, *GSTIV*, *GSTVI*, *GST21*, *GST7*, *GST37*, *ABC* and *UGT83A1* showed no significant change in response to Clo treatment (**Figures 6D, E, H–K, M–P**). However, the expression of these ten genes significantly high increased by 16.70-, 46.92-, 0.81-, 7.53-, 5.10-, 238.82-, 143.50-, 4.58-, 5.67-, 10.47-fold after CSA+Clo treatment compared to that in the Clo treatment. The expression of the GST4 gene was significantly increased by 1.68 times under Clo treatment, while it was increased by 3.97 times under CSA+Clo treatment (**Figure 6A**). Conversely, the expression of the *GST19*, *GST25*, *GST39*, *GSTZ5*, *In2-1* and *UGT76C2* genes showed a significant decrease after Clo treatment, with reductions of 75.32%, 97.09%, 39.17%, 71.26%, 78.56% and 68.90%, respectively (**Figures 6B, C, F, G, L, Q**). However, when treated with CSA+Clo, there was a notable increase in gene expression compared to Clo treatment: a fold increase of 1.71 for *GST19*, 31.51 for *GST25*, 0.87 for *GST39*, 1.95 for *GSTZ5*, 39.30 for *In2-1* and 4.20-fold increase for *UGT76C2*. The gene expression analysis revealed an up-regulation of genes related to Clo detoxification pathways in maize pre-treatment with CSA.

## 4 Discussion

The findings of our study showed that CSA effectively mitigated the adverse effects of Clo on maize seedlings, as evidenced by improvements in plant height and fresh weight (**Figure 1**). Additionally, it alleviated the decrease in chlorophyll and carotenoid content (**Figure 2**). This finding is consistent with the results of previous studies which demonstrated that CSA was able to alleviate the residual damage caused by nicosulfuron (Zheng et al., 2015). The carotenoid and chlorophyll contents in maize plants are increased when pre-treatment with CSA, even when the MEP (methylerythritol 4-phosphate) pathway is inhibited by Clo. Reactive oxygen species (ROS) are associated with herbicide toxicity, leading to membrane damage through lipid peroxidation (Gill and Tuteja, 2010). Previous studies indicated that when herbicides are absorbed by crops, the activity of SOD in leaves increases, maintaining the levels of reactive oxygen free radicals at a relatively low level. When it is not sufficient to clear the superoxide anions caused by herbicide toxicity, the SOD activity decreases and the accumulation of oxidative substances increases, which may cause damage to the cell membrane system (Xu et al., 2018). This study showed that Clo significantly elevated MDA levels while reducing SOD and CAT activities, as it can cause serious harm to maize seedlings. On the contrary, pre-treatment with CSA enhanced Clo-induced oxidative stress and reduced MDA accumulation in maize seedlings. However, CSA alone did not elevate SOD and CAT levels. This suggested that the defensive antioxidative properties are also one manner in which CSA protects maize from injury caused by Clo residue. In a previous study examining the effects of chloroacetamide herbicides and safeners (mefenpyr and dichlormid) on human blood cells, it was found that while the safeners alone did not cause any changes in oxidative stress, they did reduce the lipid peroxidation induced by the herbicides (Bernasinska et al., 2013).

The induction of glutathione S-transferases (GSTs) by safener plays a crucial role in the phase II detoxification system (Riechers et al., 2010; Baek et al., 2019). Chronopoulou et al. (2017) observed a strong correlation between the efficacy of safeners and their capacity to induce GST activity. The present study demonstrated that Clo alone or co-application with CSA was reduces GST activity GSH and GSSG content compared to CK. Pre-treatment with CSA for two hours, the GST activity and GSSG content significantly increased, this indicate that the GST enzyme facilitates the binding of GSH with the herbicide Clo, resulting in the formation of the CLO-GSH conjugate and an increase in the oxidation state of GSSG. Similarly, the safener isoxadifen-ethyl has been shown to improve maize tolerance to the toxic effects of nicosulfuron by increasing the activity of this enzyme in maize (Ye et al., 2019). Similar findings have been reported in other species. The study by Hu et al. (2020) showed that fenclorim enhances rice crop protection against pretilachlor herbicide by increasing GST enzyme activity. The results of this study, along with previous findings, suggest that CSA can enhance GST activity and mitigate herbicide-induced damage.

The molecular mechanism of safener may involve complex interactions among multiple pathways that protect plants from herbicides and other compounds (Riechers et al., 2010). Quantitative reverse transcription polymerase chain reaction (qRT-PCR) analysis was employed to examine the gene expression of detoxification enzymes, including P450, GST and UGT, in response to CSA alone or in combination with Clo treatment (**Figures 5**, **6**). Cytochrome P450 monooxygenase plays a role in the metabolism of herbicides. In this study, the expression of five genes, namely *CYP81A9*, *CYP81A4*, *CYP81A36*, *CYP72A5*, and *CYP81Q32*, was no effect or significantly down-regulated by Clo residue treatment, but their expression were significantly enhanced by the pre-treatment with CSA (**Figure 5**). These five *P450* genes may be related to metabolism of Clo. Among them, *CYP81A9* plays a crucial role in the hydroxylation process of CSA (Giannakopoulos et al., 2020) and detoxification of nicosulfuron in maize (Choe and Williams, 2020). *CYP81A4* has the potential to limit the activity of bentazon thiadiazine (Brazier-Hicks et al., 2022). This suggested *CYP81A9* and *CYP81A4* may be involved in the metabolism of Clo, but CSA itself can also be metabolized by P450.

The second phase of herbicide metabolism is primarily catalyzed by two crucial enzyme families, GSTs and UGTS (Brazier-Hicks et al., 2018). Nine *GST* genes (*GST30*, *GST31*, *GSTIV*, *GSTVI*, *GST21*, *GST7*, *GST37, GST25*, IN2-1), and two *UGT* genes (*UGT76C2, UGT83A1*) significantly high increased by 6.74-, 10.27-, 4.98-, 10.56-, 25.67-, 16.70-, 46.92-,7.53-, 5.10-, 238.82-, 143.50-, 4.58-, 31.51-, 39.3-, 4.20-, 10.47-fold after CSA+Clo treatment compared to that in the Clo treatment. Clo down-regulated the expression of these nine *GST* genes and two *UGT* genes. These results indicated that CSA and Clo induce different kinds of metabolic enzyme-encoding genes. Similar results have been reported in previous studies. Sun et al. (2018) reported that the expression levels of *ZmGSTIV*, *ZmGST6*, and *ZmGST31* can be induced by isoxadifen-ethyl alone or in combination with nicosulfuron, but are down-regulated by nicosulfuron. The study by Zhao et al. (2023) showed that the safener isoxadifen-ethyl hydrolysate induced *GSTU6* and *DIMBOA UGT BX8* gene expression in rice. The metabolism of Clo appears to be influenced by five P450 genes, nine GST genes, and two UGT genes. Further investigation is required to fully understand the role of these genes in metabolizing Clo.

## 5 Conclusions

The findings of this study confirm that CSA can reduce the residual damage caused by Clo on maize seedlings. The soaking treatment with CSA significantly increased in chlorophyll, carotenoid and GSSG contents, reduced MDA content, and enhanced SOD and GST enzyme activities in maize seedlings compared to the Clo treatment alone. Moreover, the expression of five *P450* genes (*CYP72A5*, *CYP81A4, CYP81Q32, CYP81A9, CYP81A36*), nine *GST* genes (*GST30*, *GST31*, *GSTIV*, *GSTVI*, *GST21*, *GST7*, *GST37, GST25*, IN2-1), and two *UGT* genes (*UGT76C2, UGT83A1*) significantly high increased after CSA+Clo treatment compared to that in the Clo treatment. The pre-treatment of CSA increased the expression of 5 *P450* genes, 9 *GST* genes, and 2 *UGT* genes, potentially involved in Clo metabolism in maize. The aforementioned findings provide new insights into how CSA can help reduce residual damage caused by Clo through the chlorophyll, glutathione cycle, and antioxidant enzyme activities. The expression of key genes in the glutathione metabolic pathway significantly increased under soaking with CSA. These discoveries are expected to significantly contribute to understanding the mechanism of CSA. Nevertheless, the study is subject to several constraints arising from the experimental conditions. For example, the GST enzyme activities were detected, but the potential GST-Clo metabolites could not be determined. Additionally, our study solely focused on the physiological effects of CSA on the maize shoot, without examining its impact on the root. Therefore, the findings are subject to certain limitations. Furthermore, the candidate genes identified for Clo metabolism will by functionally validated in our future work. The intention is to establish future collaborations with researchers in order to effectively address the aforementioned limitations.

## Data Availability

The original contributions presented in the study are included in the article/**Supplementary Material**. Further inquiries can be directed to the corresponding authors.
