# Supplementary material for: Efficacy and mechanism of cyprosulfamide in alleviating the phytotoxicity of clomazone residue on maize seedling
Source: Front Plant Sci. 2024 Dec 20;15:1512055. doi: 10.3389/fpls.2024.1512055 (PMC11695230; doi:10.3389/fpls.2024.1512055)
Supplement: Supplementary file 1 [file DataSheet1.docx]

1.Screening of Clomazone concentrations

Maize seeds of uniform size and fullness were selectedand subjected to a two-hour soaking process in deionised water at 25℃. Following this, the seeds were washed three times with distilled water and then spread on trays containing moistened two-layer filter paper. The trays were then placed in a light incubator. The seeds were incubated in order to facilitate germination until the emergence of the buds reached a length of 0.5 cm (28℃, photoperiod of 12D/12L, light intensity of 12,000 lx, and relative humidity of 70％).

The same size of dewy maize seeds were sown in square nutrient pots (top and bottom aperture 7 cm, 5 cm, height 7.8 cm). For each pot of 5 seeds, the soil was supplemented with clomazone (0.25, 0.5, 1, 2 and 4 mg/kg) in the clomazone treatment group and with water in the control group. Seeds were sown at a depth of 3 cm. All pots were incubated in an artificial greenhouse (temperature of 20-25℃, L//D = 12 h//12 h and 80% relative humidity). Each group of treatments was replicated four times in a completely randomised design. Plant height and chlorophyll content were measured 7 days after seedling emergence. Chlorophyll content was determined using a chlorophyll content meter (SPAD-502 PLUS, Minolta).

The results in Table 1 showed that the inhibition of plant height was 15.46%, 16.42% and 19.12%, and the inhibition of chlorophyll was 16.88%, 50.55% and 80.28% at clomazone concentrations of 1, 2 and 4 mg/kg, respectively. It can be seen that clomazone residues severely inhibited maize growth, which is similar to the results of previous studies(William K. Vencill, 1989). Maize is more sensitive to high concentrations of clomazone, so the choice of 4 mg/kg clomazone concentration is more meaningful for the study.

Table 1 Effect of different concentrations of Clomazone on the height of maize plants

| treatment | Dosage/mg∙kg^-1^ | plant height/cm | plant height inhibition rate/% | chlorophyll content/SPAD | chlorophyll content inhibition rate/% |
| --- | --- | --- | --- | --- | --- |
| Clomazone | ck | (24.13±0.83)a |  | (34.14±1.00)a |  |
|  | 0.25 | (24.10± 0.20)a | 0.12 | (32.41±0.79)a | 5.80 |
|  | 0.5 | (21.92± 0.31)ab | 9.17 | (32.87±0.84)a | 4.47 |
|  | 1 | (20.40±0.71)b | 15.46 | (28.6±0.79)b | 16.88 |
|  | 2 | (20.17±1.13)b | 16.42 | (17.01±1.22)c | 50.55 |
|  | 4 | (19.52±1.15)b | 19.12 | (6.79±0.74)d | 80.28 |

Note: The data in the table are the average of three replications. Different lowercase letters following data in the same column indicate significant differences within each treatment in corn plants, respectively (p < 0.05), determined by Duncan test.

1. Concentration screening of cyprosulfamide

Maize seeds of uniform size and fullness were selected, soaked in deionised water at 25℃ for 2 h, washed three times with distilled water and then spread on trays containing moistened two-layer filter paper, and placed in a light incubator to germinate until the buds were 0.5 cm long (28℃, photoperiod of 12D/12L, light intensity of 12,000 lx, and relative humidity of 70%).

Dew-white maize seeds of the same size were soaked in different concentrations of cyprosulfamide (7.5, 15, 30, 60 and 120 ppm) for 2 h at 26.5℃, while the control and Clomazone-treated groups were soaked in distilled water under the same conditions. Then, the treated seeds were washed three times with water. Subsequently, they were sown in square nutrient pots (top and bottom calibre 7 cm, 5 cm, height 7.8 cm). For each pot of 5 seeds, Clomazone (4 mg/kg) was added to the soil in the Clomazone-treated group and the control group was mixed with water. Seeds were sown to a depth of 3 cm. All pots were incubated in an artificial greenhouse (temperature of 20-25℃, L//D = 12 h//12 h and 80% relative humidity). Each group of treatments was replicated four times in a completely randomised design. Plant height, fresh weight and chlorophyll content were measured 7 days after seedling emergence. Chlorophyll content was determined using a chlorophyll content meter (SPAD-502 PLUS).

The results in Tables 2 and 3 showed that cyprosulfamide had good mitigating effects on Clomazone drug damage, and the mitigating effects increased with increasing concentrations of cyprosulfamide. The inhibition rates of maize plant height, fresh weight and chlorophyll content were 24.08%, 33.93% and 90.79%, respectively, under the treatment of Clomazone 4 mg/kg. The inhibition rates of maize plant height, fresh weight and chlorophyll content decreased with the addition of cyprosulfamide. The best effect of 120 ppm cyprosulfamide was found in the high concentration of Clomazone treatment, which reduced the inhibition rates of plant height, fresh weight and chlorophyll content to 13.99%, 12.86% and 16.60%, respectively.

Table 2 Effects of Clomazone and different concentrations of Cyprosulfamide on plant height and fresh weight of maize plants

| treatment | Dosage  /mg∙kg^-1^+ppm | plant height/cm | plant height inhibition rate/% | Fresh mass/g | Fresh mass  inhibition rate/% |
| --- | --- | --- | --- | --- | --- |
| CK | 0 | (21.75±0.74)a |  | (0.84±0.06)a |  |
| Clomazone | 4 | (16.51±0.32)d | 24.08 | (0.56±0.02)d | 33.93 |
| Clomazone + Cyprosulfamide | 4+7.5 | (18.59±0.53)c | 14.54 | (0.66±0.01)c | 21.67 |
|  | 4+15 | (18.99±0.332)c | 12.70 | (0.72±0.02)bc | 14.88 |
|  | 4+30 | (18.71±0.4)c | 13.99 | (0.73±0.01)bc | 12.86 |
|  | 4+60 | (19.32±0.35)bc | 11.17 | (0.75±0.01)b | 10.83 |
|  | 4+120 | (20.53±0.12)ab | 5.62 | (0.83±0.00)a | 1.19 |

Note: The data in the table are the average of three replications. Different lowercase letters following data in the same column indicate significant differences within each treatment in corn plants, respectively (p < 0.05), determined by Duncan test.

Table 3 Effects of Clomazone and different concentrations of Cyprosulfamide on chlorophyll content of maize plants

| treatment | Dosage  /mg∙kg^-1^+ppm | chlorophyll content/SPAD | chlorophyll content inhibition rate/% |
| --- | --- | --- | --- |
| CK | 0 | (31.93±0.66)a |  |
| Clomazone | 4 | (2.94±0.69)e | 90.79% |
| Clomazone + Cyprosulfamide | 4+7.5 | (8.99±0.92)d | 71.84% |
|  | 4+15 | (11.97±0.92)d | 62.51% |
|  | 4+30 | (20.45±1.24)c | 35.95% |
|  | 4+60 | (25.26±1.47)b | 20.89% |
|  | 4+120 | (26.63±1.35)b | 16.60% |

Note: The data in the table are the average of three replications. Different lowercase letters following data in the same column indicate significant differences within each treatment in corn plants, respectively (p < 0.05), determined by Duncan test.

1. Expression of related genes down-regulated by the effects of Cyprosulfamide


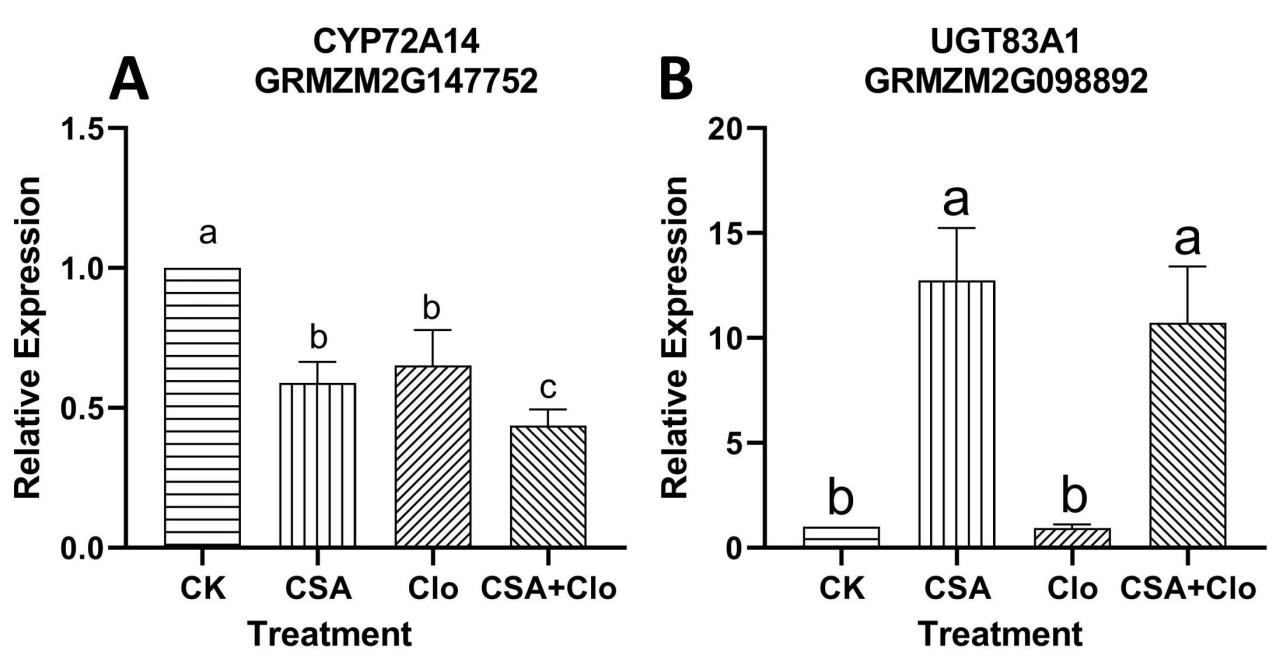


Fig. 1 (A) CYP72A14 relative expression level; ; (B) UGT88A1 relative expression level. qRT-PCR analysis were performed in three biological and two technical replicates per experiment. The bars represent mean ± SD from triplicate biological repeats. Lowercase letters indicate significant differences within each treatment in corn plants, respectively (p < 0.05), determined by Duncan test.

**References**

William K. Vencill, K.K.H.A., 1989. Growth and Physiological Responses of Normal, Dwarf, and Albino Corn (Zea mays) to Clomazone Treatments^1,2^. Pest. Biochem. Physiol. https://doi.org/https://doi.org/10.1016/0048-3575(89)90105-3.
